# Identifying the Demographic, Clinical, and Endoscopic Findings of Gastroesophageal Reflux Disease in Patients With Helicobacter pylori Infection at King Abdulaziz University Hospital, Jeddah, Saudi Arabia

**DOI:** 10.7759/cureus.26542

**Published:** 2022-07-04

**Authors:** Salem M Bazarah, Ruba M Alotaibi, Rozan A Alghamdi, Abdullah S Waheeb, Wareef A Rafeea, Sedrah K Talab, Hassan M Badawoud

**Affiliations:** 1 Department of Gastroenterology, King Abdulaziz University Hospital, Jeddah, SAU; 2 Department of Medicine, King Abdulaziz University Faculty of Medicine, Jeddah, SAU

**Keywords:** atrophic gastritis, helicobacter pylori eradication, clo test, helicobacter pylori, gastritis, gastroesophageal reflux disease (gerd)

## Abstract

Background: *Helicobacter pylori* (*H*. *pylori*) infection and gastroesophageal reflux disease (GERD) are widely spread clinical terms. GERD refers to the backflow of gastric acid to the esophagus and upper gastrointestinal tract, causing irritation. *H*. *pylori* is a gram-negative bacillus that adheres mainly to the gastric mucosa, causing peptic ulcers and gastritis. The nature of the relationship between GERD and *H*. *pylori* is yet to be explored, and few studies have been conducted. In contrast, some studies suggest a protective role of *H*. *pylori* against GERD. This study aimed to identify the demographic, clinical, and endoscopic findings of patients with GERD who underwent *H*. *pylori* testing.

Methods: A retrospective review of medical records at King Abdulaziz University Hospital, Jeddah, Saudi Arabia, between 2015 and 2021 was conducted in June 2021. Our sample consisted of 255 individuals enrolled based on age and *H*. *pylori* status. In univariate analysis, we used frequency tests for qualitative data and measure of central tendency (MCT) for quantitative data. In bivariate analysis, we used the t-test and Pearson’s chi-square test.

Results: Of 255 GERD patients enrolled, 90 were positive and 165 were negative for *H. pylori*. The majority were females (54 were positive and 93 were negative for *H. pylori*). Both groups mainly complained of abdominal pain. Endoscopically, gastritis was higher in the *H. pylori*-positive group than in the *H. pylori*-negative group.

Conclusion: In conclusion, the majority of GERD patients were *H*. *pylori*-negative, females, Saudis, and non-smokers.

## Introduction

Gastroesophageal reflux disease (GERD) and *Helicobacter pylori* (*H. pylori*) infection are common diseases worldwide [1]. GERD is one of the most prevalent upper gastrointestinal diseases globally [2]. In addition, the frequency of GERD in Saudi Arabia is slightly greater than that in western countries and significantly greater than that in East Asian nations [3]. It is a disease in which stomach contents move back into the esophagus, pharynx, larynx, or respiratory system [4]. GERD may be related to several risk factors, including obesity, smoking, and pregnancy [5], and causes acid reflux, heartburn, chest discomfort, and dysphagia, accompanied by mucosal inflammation [4]. Chronic exposure of the esophagus to gastric acid can cause several complications, such as peptic stricture, Barrett’s esophagus, and esophageal cancer [6]. *H. pylori* can be transmitted through oral-oral, fecal-oral, and gastric-oral routes [7]. The increased prevalence rates are associated with smoking, overcrowding, contaminated water, and low socioeconomic status [8,9]. Its ability to adhere, multiply, and colonize the human gastric mucosa and cause tissue damage plays a significant role in its pathogenesis [10]. Moreover, *H. pylori* is recognized as a key cause of acute/chronic gastritis and peptic ulcer disease, as well as a known causative factor for gastric cancer [4].

A cross-sectional study was performed between 2010 and 2013 at the gastrointestinal clinic of Firoozgar General Hospital, which aimed to determine the relationship between reflux disease patterns in patients with *H. pylori*, and found that the *H. pylori* infection rate was 78.1%, with a mean age of 59.8 ± 11.4 years, and 35.7% patients were males. Reflux disease was identified in 74.4% of patients [11]. In addition to another previous study [12], the results showed that the severity of GERD was reported to be lower in patients infected with *H. pylori*, indicating that *H. pylori* infection may protect against GERD. Moreover, another study has also found that *H. pylori* eradication might worsen GERD symptoms by restoring gastric acid secretion [13]. Furthermore, a prospective study conducted between 2010 and 2013 at Tor Vergata University Hospital, Rome, Italy, aimed to reveal the association between *H. pylori*-infected patients and the grade of GERD in patients with reflux, showed no statistical difference in the lower esophageal sphincter (LES) pressure among the *H. pylori*-positive and negative patients [14].

Knowledge about the demographic, clinical, and endoscopic findings of GERD in patients with *H. pylori* infection is still unclear. In our study, we aimed to identify the demographic, clinical, and endoscopic findings of GERD patients with *H. pylori* infection at King Abdulaziz University Hospital (KAUH) in Jeddah, Saudi Arabia, between 2015 and 2021.

## Materials and methods

Study design and participants

This retrospective record review study was performed in June 2021 at King Abdulaziz University Hospital (KAUH), a tertiary center in Jeddah, Saudi Arabia. The study was conducted in the Department of Internal Medicine and approved by the Institutional Review Board of KAUH (reference number: 171-20). The study was carried out according to the Good Clinical Practice (GCP) guidelines. Data were collected from the gastroenterology department’s medical electronic records between 2015 and 2021. Informed consent was waived due to the nature of the study, which was retrospective.

Data collection

The information obtained included demographic data, including age, gender, nationality, weight, and height. Clinical findings included smoking status, symptoms (fever, abdominal pain, regurgitation, heartburn, chest pain, nausea, loss of appetite, bloating, weight loss, and chronic cough), *H. pylori* status, which was detected by invasive techniques requiring endoscopy and biopsy (e.g., histological examination, culture, and rapid urease test) and by non-invasive techniques, such as serology, urea breath test, urine/blood, or detection of *H. pylori* antigen in stool specimens, comorbidities, aspirin/nonsteroidal anti-inflammatory drugs (NSAIDs) use, and proton pump inhibitor (PPI) use. Endoscopic findings obtained included gastritis, esophagitis, and hiatal hernia.

Inclusion criteria comprised patients aged > 18 years who were diagnosed with GERD and underwent testing for *H. pylori* (positive or negative) at our facility between 2015 and 2021. Patients who had only been diagnosed with *H. pylori* without GERD were excluded.

Data analysis

Microsoft Excel 2020 (Microsoft Corporation, Redmond, WA) was used for data entry. Statistical Package for the Social Sciences (SPSS) version 21 (IBM Corp., Armonk, NY) was used for statistical analysis. Data are represented as mean and standard deviation for continuous variables, and numbers and percentages for categorical variables. Student’s t-test and chi-square test were used to evaluate the differences between the continuous and categorical variables, respectively. Statistical significance was set at p < 0.05.

## Results

The present investigation aimed to identify the demographic, clinical, and endoscopic findings of GERD in patients with positive and negative *H. pylori* at KAUH, Jeddah, Saudi Arabia. A total of 661 patients were found, 255 of whom met our inclusion criteria, which are any patients who were 18 years and above diagnosed with GERD and individuals who underwent any *H. pylori* diagnostic tests. Of the patients, 90 (35.3%) were *H. pylori*-positive and the remaining 165 (64.7%) were *H. pylori*-negative.

The demographics revealed female predominance (147 out of 255, 57.6%). Among the enrolled patients, the majority were Saudi, with a total of 191 (74.9%). The mean age in our study group was 45.51 ± 15.31 years old. Regarding the classic clinical symptoms of GERD, 126 (49.4%) had abdominal pain, and 92 (36.1%) had heartburn. Of the total patients, 244 (95.7%) were non-smokers, and the majority (166, 65.1%) had no comorbidities, while 40 (15.7%) had comorbidities and 37 (14.5%) had obesity. Regarding prescribed medications, 210 (82.4%) patients used PPIs, and 87 (34.1%) used NSAIDs. In addition, the endoscopic findings included 132 (51.8%) patients who had gastritis, while 71 (27.8%) had hiatal hernia. The demographic, clinical, and endoscopic findings are summarized in Table 1.

**Table 1:** Baseline characteristics of studied patients ASA: acetylsalicylic acid; NSAIDs: nonsteroidal anti-inflammatory drugs; PPIs: proton pump inhibitors.

Variable	Value (n = 255)	Percentages
Age	45.51 ± 15.312	
Male	108	42.4%
Female	147	57.6%
Saudi	191	74.9%
Non-Saudi	64	25.1%
Smokers	11	4.3%
Comorbidities	89	34.9%
NSAIDs/ASA	87	34.1%
PPI	210	82.4%
BMI	28.36 ± 7.58	

The mean age between the two groups, i.e., *H. pylori*-positive and *H. pylori*-negative, was relatively similar (45.53 ± 15.76 and 45.5 ± 15.11, respectively). In addition, a higher number of females were *H. pylori*-positive (54, 60%), and females constituted the majority of the *H. pylori*-negative group (93, 56.3%). Also, the majority of the participants were Saudis in both groups, and around one-third of the *H. pylori*-positive were overweight (36, 40%). Apart from that, nearly half of the non-*H. pylori* participants were obese (60, 36.4%). Furthermore, eight *H. pylori*-negative and three *H. pylori-*positive participants were smokers. For their comorbidities, 12 of the *H. pylori*-positive participants were diabetic. On the other hand, the diabetic participants were greater in *H. pylori*-negative group (28).

Regarding the symptoms present in *H. pylori*-negative participants, abdominal pain (86, 68.3%) and heartburn (60, 65.2%) were two times higher than those in the *H. pylori*-positive group. For the participants who used medication, 76 (36.2%) of the *H. pylori*-positive participants were using PPIs, and only 32 (36.8%) used NSAIDs. Furthermore, the number of *H. pylori*-negative participants who used PPIs was 134 (63.8%) and 55 (63.2%) for those who used NSAIDs. Along with those who underwent endoscopy, 53 (40.31%) of the *H. pylori*-positive participants had gastritis, while 79 (59.8%) of the *H. pylori*-negative participants developed gastritis. Moreover, endoscopic findings also showed that 23 (37.1%) patients with *H. pylori* had signs of esophagitis, while only 39 (62.9%) patients without *H. pylori* had esophagitis. In the *H. pylori*-negative group, 49 (69%) had hiatal hernia, while 22 (30.98%) of those who were *H. pylori*-positive had hiatal hernia, as shown in Tables 2, 3.

**Table 2:** Bivariate statistics for demographic parameters ASA: acetylsalicylic acid; NSAIDs: nonsteroidal anti-inflammatory drugs; PPIs: proton pump inhibitors.

Variables	Positive *Helicobacter pylori* (n = 90)	Negative *Helicobacter pylori* (n = 165)	P-value
Age	16.46 ± 16.46	45.50 ± 15.11	0.827
BMI	27.78 ± 6.32	28.68 ± 8.19	0.33
Male, n = 108	36 (33.3%)	72 (66.7%)	0.668
Female, n = 147	54 (60%)	93 (56.3%)	0.668
Saudi	68 (75.6%)	123 (74.5%)	0.777
Non-Saudi	22 (24.4%)	42 (25.5%)	0.777
Smoking status, n = 11	3 (27.3%)	11 (72.7%)	0.751
Comorbidities, n = 89	26 (29.2%)	63 (70.8%)	0.359
PPI, n = 210	76 (36.2%)	134 (63.8%)	0.635
NSAIDs/ASA, n = 87	32 (36.8%)	55 (63.2%)	0.826

**Table 3:** Bivariate statistics for clinical parameters ASA: acetylsalicylic acid; NSAIDs: nonsteroidal anti-inflammatory drugs; PPIs: proton pump inhibitors.

Variables	Positive *Helicobacter pylori* (n = 90)	Negative *Helicobacter pylori* (n = 165)	P-value
Abdominal pain, n = 126	40 (31.7%)	86 (68.3%)	0.298
Fever, n = 6	1 (16.7%)	5 (83.3%)	0.429
Regurgitation, n = 73	24 (32.9%)	49 (67.1%)	0.714
Heartburn, n = 92	32 (34.8%)	60 (65.2%)	1
Chest pain, n = 25	10 (40%)	15 (60%)	0.766
Nausea, n = 51	15 (29.4%)	36 (70.6%)	0.413
Loss of appetite, n = 6	2 (33.3%)	4 (66.7%)	1
Bloating, n = 38	14 (36.8%)	24 (63.2%)	0.974
Weight loss, n = 10	4 (40%)	6 (60%)	0.745
Chronic cough, n = 12	3 (25%)	9 (75%)	0.548
Endoscopic and pharmacologic			
Gastritis, n = 132	53 (40.2%)	79 (59.8%)	0.121
Esophagitis, n = 62	23 (37.1%)	39 (62.9%)	0.85
Hiatal hernia, n = 71	22 (30.98%)	49 (69.0%)	0.454
Drug use			
PPI, n = 210	76 (36.2%)	134 (63.8%)	0.635
NSAIDs/ASA, n = 87	32 (36.8%)	55 (63.2%)	0.826

## Discussion

This study was conducted to explore the demographic, clinical, and endoscopic findings of GERD in patients who underwent *H. pylori* testing. The results revealed that 35.3% of individuals were *H. pylori*-positive, and the majority of them were women (57.6%). Moreover, there were no significant differences in the demographic and clinical features between the *H. pylori*-positive and negative groups. Endoscopically, gastritis was the commonest finding, whereas the commonest drugs used by the study group were PPIs.

Previous studies suggested that the prevalence of *H. pylori*-infected patients with GERD varies from 30% to 90%, but in most cases, it is 30% [15], which is quite similar to what we discovered in our study (35.3%). This highlights the geographical variations reported in *H. pylori* infection prevalence in the Far East, North America, and Western Europe [16], which is not reflected in this study since the majority were Saudis. Additionally, this variation can be due to the effects of cofactors such as genetics, diet, and methods used for *H. pylori* testing to detect early diagnosis, which differs from one region to another [16].

According to our study, 57.6% of *H. pylori*-positive patients with GERD were women. Similar results were found in another study performed in Israel, where the majority (88%) of the patients were women. Moreover, another study aimed to explore the relationship between GERD and *H. pylori* reported a high prevalence among females [17,18]. This could be due to maternity factors, such as pregnancy [17].

Regarding the epidemiological distribution, the mean age of our study group was 45.50 ± 15.57 years. A study conducted at Fort Jackson, South Carolina, regarding the age-specific prevalence, showed that only 20.8% of patients were in the 15-24 years age group. In contrast, previous studies have reported that *H. pylori* infection was detected earlier in the younger population in many developing countries, which reflects the variation in age distribution in earlier studies and the socioeconomic status of the participants [19].

In addition, our results show that Saudi patients have a higher risk of developing GERD and *H. pylori* than non-Saudis, which might be a result of having the data collected only from KAUH in the Kingdom of Saudi Arabia. In addition, we found another Israeli study that showed that Arabs have a higher risk of acquiring *H. pylori* infection along with GERD [17].

The present study showed that abdominal pain was a common clinical manifestation for both groups who had GERD and developed or did not develop *H. pylori*. In addition, another study aimed to discover the association between GERD and *H. pylori* revealed that most of the patients with GERD also complained of abdominal pain. However, the majority were not infected with *H. pylori* (94.1%), while only 5.9% were infected [20]. This could be a result of inflammatory mediators that were activated in patients with GERD. The initial thought stated that *H. pylori* was protective against GERD through the impairment of gastric acidity, which seems consistent with our results; therefore, the *H. pylori* negatives were found to have a higher incidence of GERD [17].

Our data showed a higher proportion of *H. pylori* among non-smokers (87, 96.67%; p = 0.751), although the association between smoking and *H. pylori* was not significant. Interestingly, another research that was carried out among smokers stated that *H. pylori* is inversely related to dose-dependent cigarette consumption, which means that heavy smokers tend to be less exposed to *H. pylori* infection. This may be due to the increased gastric acidity observed during smoking, resulting in the dose-dependent inverse relationship between cigarette consumption, smoking, and *H. pylori* [21]. Since the research team was using secondary data, a high number of the smoking section questions were poorly documented in the clinical setting records. Thus, this might affect our results in this term.

On the other hand, our study shows that regardless of the *H. pylori* status, most of the patients were using PPIs. It is known that long-term use contributes to a higher risk of corpus atrophy, which results in greater inhibition of gastric secretions among *H. pylori*-infected patients, unlike the uninfected ones [22], which can explain the greater inhibition of gastric acid noted in patients with *H. pylori* who were using PPIs, and lower prevalence of advanced esophagitis and GERD in *H. pylori* infection [17].

Endoscopic findings revealed that 37.1% of *H. pylori*-positive patients and 92.9% of *H. pylori*-negative patients had esophagitis. This was similar to a Turkish study that showed that patients with reflux symptoms or esophagitis had a lower prevalence of *H. pylori* infection. This could be due to atrophy of the parietal cells, in which the production of hydrochloric acid (HCl) is their normal function. Furthermore, atrophy of the parietal cells, which is caused by the microorganism in the case of *H. pylori* infection, leads to decreased HCl secretion, suggesting a protective mechanism against GERD [23].

Our study was limited by the small sample size, poor documentation, and the retrospective nature of the study. The on-ground diagnosis of GERD without pH monitoring threatens the accuracy of the results, and hence all are considered part of our limitations. Consequently, the sample size should be increased to obtain more generalized results. Moreover, a case-control study design is preferred, as it would help establish a clear relationship between the two. In addition, direct contact with patients and a clear history of the symptoms would be preferable.

## Conclusions

In conclusion, we found that most patients with GERD who tested positive for *H. pylori* were middle-aged, Saudi nationals, female, and non-smokers. The *H. pylori*-positive group tended to use more PPIs, mostly complained of abdominal pain, and showed gastritis as the most common findings. In addition, few *H. pylori*-infected patients had esophagitis. The uninfected group enrolled with GERD mainly consisted of Saudis, females, and non-smokers. Similar to the *H. pylori*-positive group, the commonest complaint was abdominal pain and gastritis. Future research efforts should focus on large-scale population-based studies and deciphering the pathogenesis of GERD in *H. pylori*-positive versus negative patients.
